# Entrepreneurial Passion Psychology- Based Influencing Factors of New Venture Performance

**DOI:** 10.3389/fpsyg.2021.696963

**Published:** 2021-08-10

**Authors:** Shouzheng Zhao, Yuqin Zhou, Hai Guan, Wenhai Xu

**Affiliations:** ^1^School of Law, Shanghai International Studies University, Shanghai, China; ^2^School of Law, Tongji University, Shanghai, China

**Keywords:** entrepreneurial passion, new venture performance, legal risk, work engagement, entrepreneurs

## Abstract

Small and medium-sized enterprises have been the driving force of social economy. As the social formation and the rise of many emerging economies, the domestic economic environment changes dramatically, which makes small and medium-sized enterprises face severe challenges. In the face of the fierce competition environment, how to improve the performance of entrepreneurship and gain competitive advantage is a very important topic in the practical management of entrepreneurs. This exploration is carried out with the entrepreneurial passion and risk taking as the antecedent variable, entrepreneurial performance as the outcome variable, and then work engagement as the mediator. With the entrepreneurs of small and medium-sized enterprises as the research object, questionnaire method is used to carry out empirical research. The empirical analysis results show that entrepreneurial passion has a significant positive impact on work engagement; risk taking has a significant positive impact on work engagement; work engagement has a significant positive impact on entrepreneurship performance; in addition, work participation plays a mediating role in the process of entrepreneurial passion and risk taking. The results provide academic and practical implications for entrepreneurs.

## Introduction

In recent years, governments all over the world encourage entrepreneurship. Entrepreneurship research has become a fast-growing and increasingly important research in global enterprise research. Therefore, the business trends of small and medium-sized enterprises deserve attention (Aldrich, 2017). Especially in the social formation and the rise of many emerging economies, the domestic economic environment changes dramatically, which makes small and medium-sized enterprises face severe challenges. In the face of fierce competition, how to maintain entrepreneurial performance and occupy a place in the market is a very important management topic for entrepreneurs in practical operation (Wadhwani et al., 2020). Small and medium-sized enterprises are very important for the improvement of economic level, which is conducive to the further development of the national economy. The survey results suggest that with the change of market environment, the proportion of small and medium-sized enterprises in the whole economic system has gradually increased. By 2020, they have contributed nearly 70% of GDP (Bhatt et al., 2019). Small and medium-sized enterprises and new ventures can create more employment opportunities for the society than large enterprises, and the main role of driving the country’s economic growth and leap forward is the entrepreneurs with entrepreneurial ability and innovation vitality.

The prosperity of entrepreneurship can make up for the deficiencies of unemployment rate and maintain a stable state of economic balance. Entrepreneurial activities contribute a lot to social and economic growth (Obschonka et al., 2019a). Entrepreneurship timely provides another way to choose; in addition, in recent years, with the rapid development of science and technology, the rise of Alibaba has accelerated the trend of entrepreneurship; aspiring people have joined the ranks of entrepreneurship, want to rely on their own unique ideas and creativity, give play to entrepreneurial passion and action, expect to get feedback from the market, and develop into a successful business under the refined trial mode (Howell, 2019). However, it is difficult to create business and keep success. From the perspective of business management, both are difficult. Therefore, how to maintain entrepreneurial performance is worth discussing.

Passion needs to be maintained in the participation of any entrepreneurial activities. Only with entrepreneurial passion can entrepreneurs maintain sensitivity to the neglected needs in the surrounding environment and further reflect on the behavior. One of the research motivations is to understand the impact of entrepreneurial passion on entrepreneurial performance. Entrepreneurs are not all risk seekers as people all know. They are not entirely seeking high-risk businesses, but they are more willing to bear the risks that new businesses should have and reasonably estimated than others. The second motivation of this exploration is to explore the impact of risk taking on entrepreneurial performance, in order to understand whether the degree of uncertain risk taking of entrepreneurs will affect the business performance of enterprises. The influence of entrepreneurial passion, risk taking, work engagement and entrepreneurial performance is mainly discussed. From the perspective of entrepreneurial passion, the influencing factors of entrepreneurial performance of new ventures, and the strategic choices of new ventures when facing legal risks are discussed. The research innovation is to evaluate the relevant factors that affect the new enterprise performance through the relevant theories of entrepreneurial passion psychology, and get the relevant influencing factors by verifying the hypothesis. It is hoped that this exploration can optimize the management of enterprises and improve the enthusiasm of employees.

## Materials and Methods

### Entrepreneurship and Entrepreneurial Passion

Entrepreneurship is to create a new business of their own from nothing. It contains the meaning of creation, and also contains three main characteristics: (1) creativity and innovation; (2) the establishment of the combination of economic organizations and resources; (3) under the environment of uncertain risks, identify the opportunity and ability of growth, and then create a new economic organization. Entrepreneurship needs passion, because passion is the driving force behind entrepreneurship and the key to success (Gielnik et al., 2017). Entrepreneurial passion is more challenging and requires more wisdom than general activity passion, although it is often accompanied by pressure.

Warnick et al. pointed out that passion has long been recognized as an important component of entrepreneurial motivation and successful combination. Passion is at the heart of entrepreneurship because it promotes creativity and patterns of identifying new information (Warnick et al., 2018). Milanesi defined entrepreneurial passion as a positive and strong emotional state of an entrepreneur, accompanied by cognitive and personal high-value behavior (Milanesi, 2018). Schenkel et al. believed that entrepreneurship is the embodiment of passion, and entrepreneurial passion is a positive emotion with creativity and sustainability (Schenkel et al., 2019). Stroe et al. pointed out that entrepreneurial passion is a strong and positive emotion, and it is also a conscious approach, feeling positive emotion in self-identity activities (Stroe et al., 2018). Arshad et al. proposed that passion can be the driving force of entrepreneurial action (Arshad et al., 2018). In the study of the relationship between entrepreneurial passion and business performance, Guercini and Ceccarelli defined entrepreneurial passion as a perceptible and powerful positive emotional state, accompanied by the display of cognition and behavior that are meaningful to the role of entrepreneurs and can highlight self-identity and high personal or organizational value (Guercini and Ceccarelli, 2020). Wu and Wu studied the impact of positive and negative emotions of enterprise supervision on employees’ innovation behavior, and on this basis, studied the mediating role of work participation and demonstration behavior under this path (Wu and Wu, 2019).

Based on the above literature discussion, entrepreneurial passion is that individuals are willing to invest a lot of time and effort in entrepreneurial activities. Through internalizing self-identity, they can stimulate creativity and generate persistent strong positive emotions. With the entrepreneurs of small and medium-sized enterprises as the main research object, their initial passion is discussed, and the entrepreneurial passion is divided into two levels: the starting passion and the founding passion.

### Entrepreneurial Performance of New Ventures

The goal of all the management and strategy of an enterprise is to create profit, and expect to maximize profits by minimizing resources. Gao et al. believed that performance is the concept of integrity that the enterprise presents the final results of the operation activities, and also measures the achievement degree of the enterprise’s objectives (Gao et al., 2018). Eniola et al. pointed out that enterprise performance is a measure of the extent to which an enterprise aims to achieve its long and short-term business goals (Eniola et al., 2019). Dong et al. proposed that entrepreneurship performance is the satisfaction of entrepreneurs with their own entrepreneurial results, and can also be regarded as the basic method to measure performance (Dong et al., 2020).

Del Giudice et al. divided performance into three categories: financial performance, career performance and organizational effectiveness (Del Giudice et al., 2017). Spanjer and van Witteloostuijn pointed out that organizational performance can be measured by market share, sales level, cost control, profitability and overall performance (Spanjer and van Witteloostuijn, 2017). Ju et al. divided entrepreneurial performance into two categories: financial performance indicators, that is, indicators are presented in figures (Ju et al., 2019). Wu et al. pointed out that entrepreneurial performance should include overall satisfaction and financial satisfaction. The former is an important indicator to test the marketing activities of enterprises, while the latter is the comparison of the actual sales and profits of the company with the expectations before the start-up (Wu et al., 2020).

Entrepreneurial performance is an important operating indicator of an enterprise, and how to create excellent entrepreneurial performance has become an important issue for entrepreneurs in management. Entrepreneurial performance is defined as the measure of the entrepreneur’s satisfaction with the achievement rate of the enterprise’s expected goals and operating results. With entrepreneurs with entrepreneurial experience as the main research object, entrepreneurial performance is divided into economic indicators and non-economic indicators.

### Risk Taking of New Ventures

With the increasing scale and variety of enterprises, there are more and more challenges in risk management, which make risk management become a new systematic discipline at this time. The classification of different risks faced by enterprises is also refined, and the research on legal risk management begins (Akbar et al., 2017). In recent years, scholars have done more research on enterprise legal risk management from a micro perspective, and the overall research tends to be differentiated.

Díez-Esteban et al. divided the legal risks faced by enterprises into three categories: preventable risks, strategic risks and external risks. They pointed out that enterprises should adopt different types of management methods for different types of legal risks, which will make risk management more effective (Díez-Esteban et al., 2019a). Shahzad et al. believed that enterprises should actively manage the legal risks of electronic transactions. Furthermore, the strategy analysis of legal risk management in the process of electronic transaction by enterprises as sellers or service providers was discussed (Shahzad et al., 2019). Yost thought that risk taking refers to the degree of tendency of managers to make bold and risky commitment to all resources. Risk taking is an enterprise willing to take on the assessed risk in order to pursue an appropriate opportunity (Yost, 2018). Ferris et al. proposed that risk taking refers to the degree of willingness to invest a lot of resources in uncertainty and new ventures (Ferris et al., 2019). Gupta and Krishnamurti’s interpretation of risk taking refers to the tendency of individuals to avoid or take risks when they encounter risks in different situations (Gupta and Krishnamurti, 2018). Lee et al. discussed the relationship among corporate governance, risk taking and business performance. The results suggest that corporate ownership is positively correlated with corporate risk-taking, and has a significant impact on corporate performance (Lee et al., 2021). Ljungqvist et al. explored the factors that influence entrepreneurial success. The results show that risk taking, innovation and anticipation in entrepreneurial strategy have a significant impact on entrepreneurial success (Ljungqvist et al., 2017).

The literature discussion and related research on risk taking by scholars in China and foreign countries reveal that most people think that risk taking comes from the judgment of individual external stimulus value in daily life, which leads to risk judgment and risk evaluation. This exploration aims to explore the relationship between risk taking and entrepreneurial performance. In the process of entrepreneurship, the degree of entrepreneurs’ willingness to take risks is one of the key factors that affect entrepreneurial performance.

### Work Engagement of Employees in New Ventures

In the process of enterprise management, whether employees can devote themselves to their work to maximize their effectiveness is the most concerned problem of managers. The concept of work engagement is first put forward based on self-engagement and the focus of interest in life, and extended according to its meaning in work: (1) it is the degree of personal psychological identification with work and the importance of personal self-impression; (2) It is the degree of personal active participation in work and the degree of personal self-esteem affected by work performance (Bakker and Leiter, 2017; Díez-Esteban et al., 2019b).

Knight et al. proposed that work engagement refers to the degree of personal psychological recognition of work and the importance of cognitive work performance to self-worth (Knight et al., 2017). Gürlek and Tuna said that work engagement is regarded as an individual’s psychological commitment or recognition to work (Gürlek and Tuna, 2019). Amor defined work engagement as the degree of personal psychological recognition of the work and its position and explicit behavior attitude, and identification with one’s own work performance has an important impact on self-worth (Amor et al., 2020). Cesário and Chambel believed that work engagement is a kind of persistent characteristic, full of positive feeling and strong motivation for work, which contains three characteristics: vitality, dedication and concentration (Cesário and Chambel, 2017). Orgambídez-Ramos and de Almeida explored the influence of personality traits on work engagement and work performance. The results reveal that work engagement has a significant positive correlation with work performance; personality traits also indirectly improve work performance through the improvement of work engagement (Orgambídez-Ramos and de Almeida, 2017). van Mol et al. explored the correlation between personality traits and work engagement. The results suggest that there is a significant positive correlation between personality traits and work engagement (van Mol et al., 2018). De Simone et al. explored the relationship among personality traits, work engagement, emotional labor and work performance. The results show that the higher the employee’s work engagement is, the better the work performance is, while the open personality, affinity personality and work engagement have a positive and significant impact (De Simone et al., 2018). Obschonka et al. studied the influence process of entrepreneurial passion in the aggregation of organizational behavior, and obtained a people-oriented and variable-oriented trait approach through quantitative analysis of the influence of personality traits on entrepreneurial passion in the organization. The results show that the entrepreneurs’ basic entrepreneurial personality helps to shape their entrepreneurial passion (Obschonka et al., 2019b).

Based on the above domestic and foreign scholars and researchers’ relevant theories and literature discussion on work engagement, this exploration defines work engagement as a kind of emotion and attitude, and has the characteristics of persistence. It means to actively participate in the current work, continuously maintain a strong motivation, and affirm oneself with achievements. Based on the above research level, the work engagement is divided into three levels: vitality, dedication and concentration.

### A Model of Influencing Factors on the Performance of New Ventures

**FIGURE 1:**
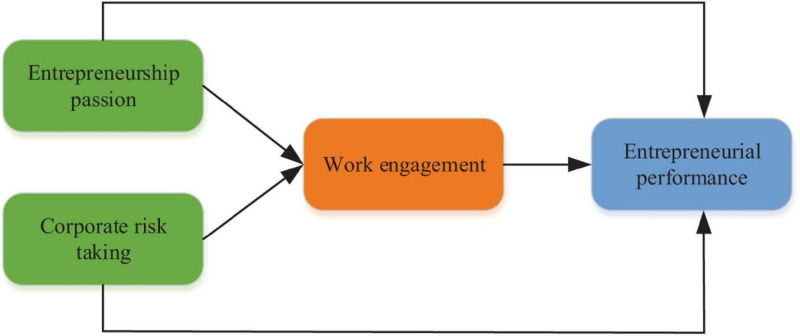
Diagram of relationship structure of key variables.

The research focus is to understand the relationship among entrepreneurial passion, risk taking, work engagement and entrepreneurial performance, and whether entrepreneurial passion and risk taking can affect entrepreneurial performance through the mediating effect of work engagement. Figure 1 is the framework of this exploration.

This exploration indirectly infers from the research on the relationship between personality traits and entrepreneurial performance, because passion is a part of personality traits, and the discussion of personality traits also includes empirical research on passion. There are few studies on the relationship between risk taking and entrepreneurial performance. This exploration is based on the relevant research of other industries. It is found that the higher the degree of financial risk voluntary commitment of employees in the investment department is, the higher the investment performance of the company is. The higher the risk is, the higher the reward is. The more employees in the investment department dare to take financial risks, the higher their reward investment performance will be. There are few studies on the relationship between work engagement and entrepreneurial performance.

However, if the entrepreneurial performance is regarded as the business performance of the organization, the results of the study on the organizational performance also confirm that the work engagement will have a positive impact on the performance. Since passion is one of the five personality traits, this exploration uses the relationship between personality traits and work engagement to infer the hypothesis. The influencing factor model and hypothesis with work engagement as the intermediary variable are set up. In order to make a more accurate research on the relationship among different influencing factors, the hypotheses and sub hypotheses are put forward as shown in Table 1 by combining the references in relevant research fields. The proposed assumptions are verified to determine the relationship among the influencing factors.

**TABLE 1:** Research hypotheses.

**Hypotheses**	**Content**
H1:	There is a significant positive relationship between entrepreneurial passion and entrepreneurial performance.
H1-1:	Starting passion has a positive and significant impact on the economic indicators of entrepreneurship performance.
H1-2:	Founding passion has a positive and significant influence on the economic indicators of the entrepreneurial performance.
H1-3:	Starting passion has a positive and significant impact on the non-economic indicators of entrepreneurial performance.
H1-4:	Founding passion has a positive and significant influence on the non-economic indicators of the entrepreneurial performance.
H2:	There is a significant positive relationship between risk taking and entrepreneurial performance.
H2-1:	Risk taking has a positive significant impact on the economic indicators of entrepreneurial performance.
H2-2:	Risk taking has a positive significant impact on the non-economic indicators of entrepreneurial performance
H3:	There is a significant positive relationship between work engagement and entrepreneurial performance.
H3-1:	Vitality has a significant positive impact on the economic indicators of entrepreneurial performance.
H3-2:	Dedication has a positive and significant impact on the economic indicators of entrepreneurial performance.
H3-3:	Concentration has a positive and significant impact on the economic indicators of entrepreneurial performance.
H3-4:	Vitality has a significant positive impact on the non-economic indicators of entrepreneurial performance.
H3-5:	Dedication has a positive and significant impact on the non-economic indicators of entrepreneurial performance.
H3-6:	Concentration has a positive and significant impact on the non-economic indicators of entrepreneurial performance.
H4:	There is a positive and significant relationship between entrepreneurial passion and work engagement.
H4-1:	Starting passion has a positive and significant impact on the vitality of work engagement.
H4-2:	Founding passion has a positive and significant impact on the vitality of work engagement.
H4-3:	Starting passion has a positive and significant impact on the dedication of work engagement.
H4-4:	Founding passion has a positive and significant influence on the dedication of work engagement.
H4-5:	Starting passion has a positive and significant impact on the concentration of work engagement.
H4-6:	Founding passion has a positive and significant impact on the concentration of work engagement.
H5:	There is a significant positive relationship between risk taking and work engagement.
H6:	Entrepreneurial passion indirectly affects entrepreneurial performance through the mediating effect of work engagement.

### Dimension Measurement and Survey Design of Key Variables

This exploration refers to Thorgren and Wincent’s research level and operational definition (Thorgren and Wincent, 2015), and uses the scale of entrepreneurial passion constructed by Thorgren and Wincent to develop the measurement items according to the research needs, including four questions about entrepreneurial passion and four questions about founding passion, with a total of eight questions in the questionnaire. Risk taking is defined as people’s understanding of specific risks in the process of engaging in any activity, and then determine how much risk they can bear through assessment. Based on the above operational definition, risk taking items of this exploration are established. There are four questions in the questionnaire. According to Schaufeli and Salanova’s definition of work engagement, work engagement is defined as a kind of emotion and attitude with persistent characteristics, actively participating in the current work, continuously maintaining strong motivation, and affirming oneself with achievements (Schaufeli and Salanova, 2007). According to Schaufeli and Salanova’s measurement of work engagement, there are three questions in the level of vitality, four questions in the level of dedication and four questions in the level of concentration, with a total of 11 questions. According to the research needs, the measurement items of the scale of entrepreneurial performance variables are developed, including 3 economic indicators and 4 non-economic indicators, with a total of 7 questions. Economic indicators include net income, net profit and net asset income, while non-economic indicators include team building, safety management, service quality and employee satisfaction.

The main research object of the questionnaire is entrepreneurs. The main sample source is entrepreneurs of small and medium-sized enterprises in Zhejiang Province. Among them, the average age of these entrepreneurs is between 35 and 45 years old, and the proportion of males and females is equal, and they all have bachelor’s degree or above. The questionnaire will be distributed and collected from July to September 2020. A total of 200 questionnaires are distributed and 168 are collected. Then, manual screening is conducted to deduct 18 invalid questionnaires such as incomplete answers and abnormal answers. Finally, 150 valid samples are obtained, and the effective questionnaire recovery rate is 75%. The mean variance extracted (AVE) method is used to test the discriminant validity. Cronbach’s α is used to test the reliability of the scale. The AVE value of each dimension of the scale is greater than the correlation coefficient. The calculated Cronbach’s α coefficient is 0.975. Since it is much larger than the coefficient of 0.6, the questionnaire has good reliability, which can meet the needs of this survey. The scale used has high internal consistency, good stability and overall reliability, which can meet the needs of scientific research. The contents of the questionnaire are mainly entrepreneurial passion, risk-taking and work engagement. The questionnaire uses a five-level sequential scale to measure the impact of entrepreneurial passion on entrepreneurial performance, which is divided into five levels: no impact, basically no impact, some impact, relatively impact and siginifant impact, and its assignment is 1 point, 2 points, 3 points, 4 points and 5 points. Finally, the data is processed by SPSS25.0 software.

## Results and Discussion

### Correlation Analysis of Key Variables

**FIGURE 2:**
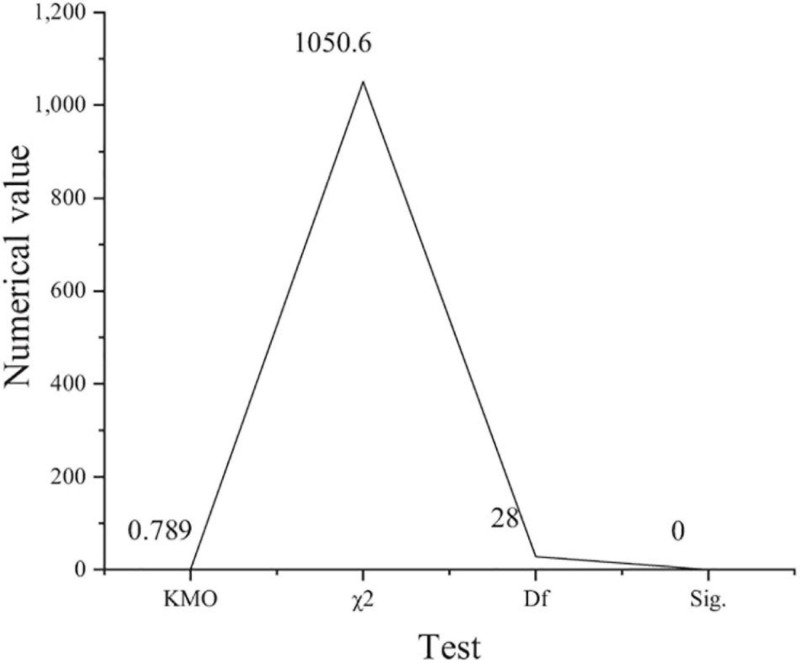
Analysis results of entrepreneurial passion model (χ^2^: approximate chi square; Df: degree of freedom; Sig: Significance level).

Figure 2 shows the results of the model feasibility analysis.

**FIGURE 3:**
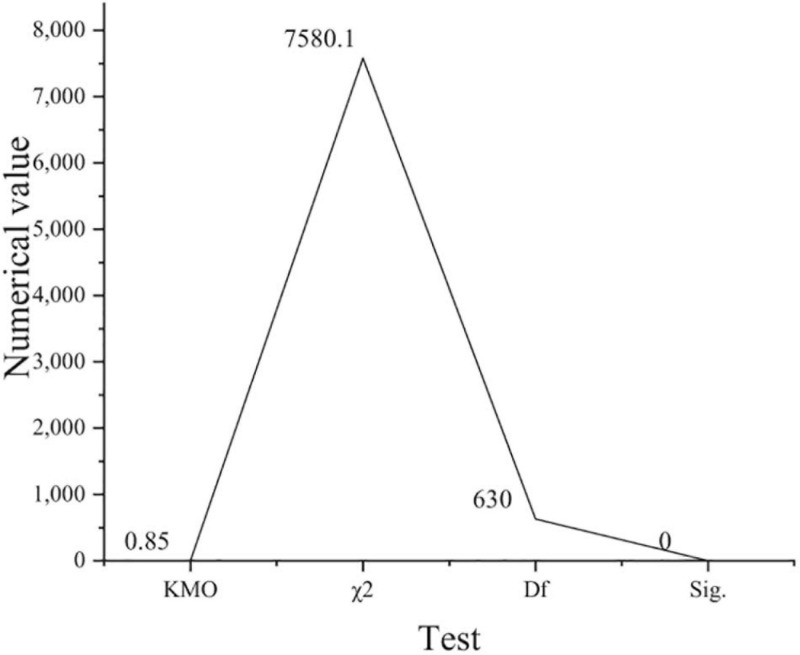
Feasibility analysis results of risk bearing model (χ^2^: approximate chi square; Df: degree of freedom; Sig: Significance level).

Figure 2 shows that KMO is 0.789, which is greater than 0.7, and Sig is 0, which is less than 0.05. The comparison of the two groups of data shows that it meets the requirements of factor analysis and meets the needs of this survey. Figure 3 shows the validity analysis of the risk-taking questionnaire.

**FIGURE 4:**
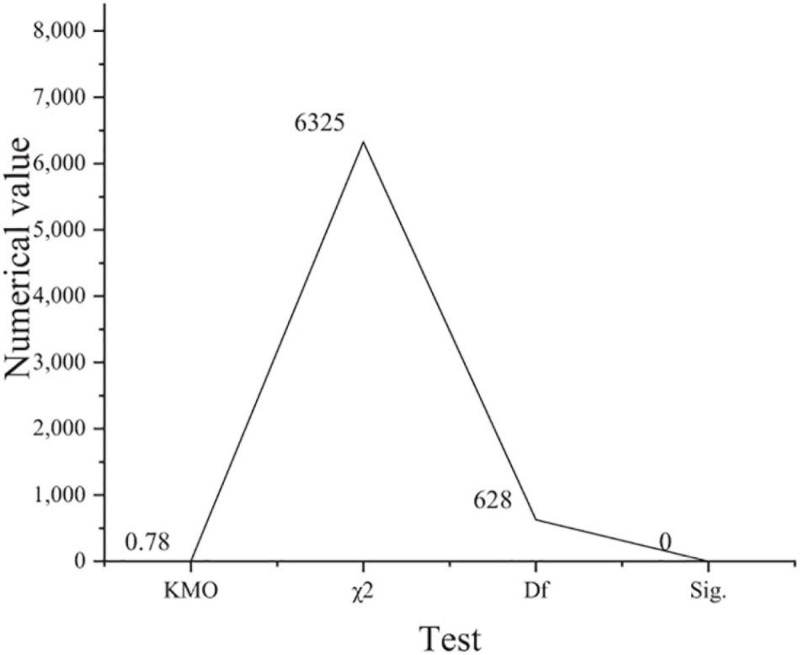
Validity analysis results of work engagement questionnaire (χ^2^: approximate chi square; Df: degree of freedom; Sig: Significance level).

Figure 3 shows that KMO is 0.85 and greater than 0.7, and Sig is 0 and less than 0.05. The comparison of the two groups of data shows that it meets the requirements of factor analysis. Figure 4 shows the feasibility analysis of the work engagement model.

Figure 4 shows that KMO is 0.78 and greater than 0.7. Sig is 0 and less than 0.05. The comparison of the two groups of data shows that it meets the requirements of factor analysis.

Table 2 shows the validity of the questionnaire.

**TABLE 2:** The validity of the questionnaire.

	**A**	**B**	**C**
A	1		
B	0.553**	1	
C	0.567**	0.632**	1

** means that here is a significant correlation at 0.1 (A, entrepreneurial passion; B, risk-taking; C, work engagement).

The data in the table above show that the variables are independent and related to each other, which shows that the questionnaire used has good structural validity.

Before regression analysis, Pearson correlation analysis is used to examine whether there is correlation between independent variables and dependent variables to determine whether the model is suitable for regression analysis. Moreover, the Pearson correlation coefficient test is used to observe whether there is a significant correlation among entrepreneurial passion, risk taking, work engagement and entrepreneurial performance and how close the relationship is. Table 3 shows the results of Pearson correlation analysis.

**TABLE 3:** Correlation analysis of variables.

**Variables**	**Average**	**Standard deviation**	**Entrepreneurial passion**	**Risk taking**	**Work engagement**	**Entrepreneurial performance**
Entrepreneurial passion	6.149	0.372	1			
Risk taking	6.193	0.582	0.543**	1		
Work engagement	6.228	0.823	0.664**	0.574**	1	
Entrepreneurial performance	6.014	0.564	0.575**	0.582**	0.595**	1

** means that at a significant level of 0.01 (double tail), it is significantly related.

According to the above table, the degree of relationship among the variables is as follows: (1) entrepreneurial passion is significantly positively correlated with risk taking, work engagement and entrepreneurial performance, with correlation coefficients of 0.543, 0.664, and 0.575, respectively; (2) risk taking is significantly positively correlated with work engagement and entrepreneurial performance, with correlation coefficients of 0.574 and 0.582, respectively; (3) work engagement and entrepreneurial performance are also significantly positively correlated, with a correlation coefficient of 0.595. There is a significant positive correlation among the variables.

### Multiple Regression Analysis of Entrepreneurial Passion and Entrepreneurial Performance

**FIGURE 5:**
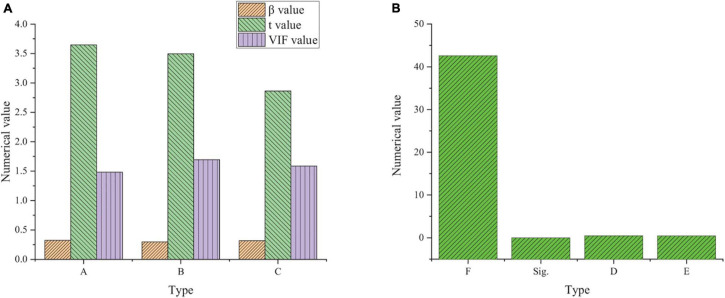
Multiple regression analysis of entrepreneurial passion, risk-taking and work engagement on entrepreneurial performance [**(A)** data analysis data; **(B)** calculation and statistics; A, Entrepreneurial passion; B, Risk taking Work; C, engagement; D, R^2^; E, △R^2^].

Multiple regression analysis is used to further explore the impact degree among entrepreneurial passion, risk taking, work engagement and entrepreneurial performance, and test the mediating effect of work engagement. The variance inflation factor (VIF) is an indicator used to measure the degree of multicollinearity in multiple linear regression models. Its value is the ratio of the variance of the regression coefficient estimator to the variance when the independent variables are not linearly correlated. In order to evaluate the effectiveness of the regression model, the VIF values of all levels are tested to determine whether there is collinearity. Figure 5 shows the test and analysis results.

The analysis results in the above table show that the critical value of VIF is less than 10, and there is no collinearity problem. The results of multiple regression analysis show that the F value of entrepreneurial passion, risk taking and work engagement on entrepreneurial performance is 42.593, the β value is 0.324, 0.296, and 0.317, respectively, the R^2^ value is 0.468, and the *P* value is 0.000, indicating that the regression model has reached a significant level, which verifies the hypothesis H1, H2, and H3.

**FIGURE 6:**
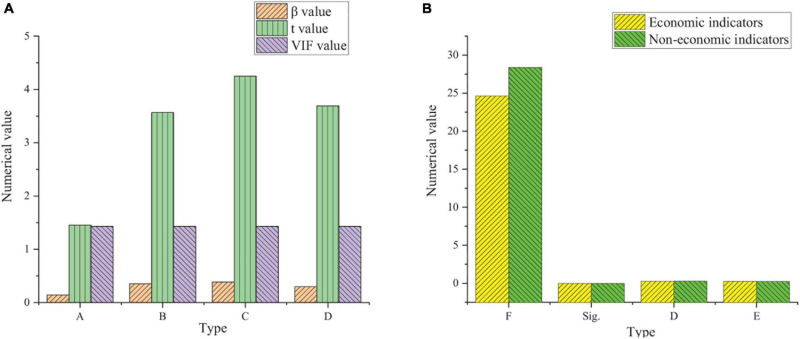
Regression Analysis of entrepreneurial passion and entrepreneurial performance at all levels [**(A)** data analysis data; **(B)** calculation and statistics; A, Entrepreneurial passion; B, Risk taking Work; C, engagement; D, R^2^; E, △R^2^].

Figure 6 shows that starting passion in entrepreneurial passion has a significant positive impact on the economic indicators of entrepreneurial performance, with F value of 24.636, β value of 0.142 and 0.353, R^2^ value of 0.274 and *P* value of 0.000. Founding passion in entrepreneurial passion has a significant positive impact on the non-economic indicators of entrepreneurial performance. The F value is 28.391, β value is 0.385, and 0.297, R^2^ value is 0.295, and *P* value is 0.000. The regression model verifies the hypotheses H1-1, H1-2, H1-3, and H1-4.

**FIGURE 7:**
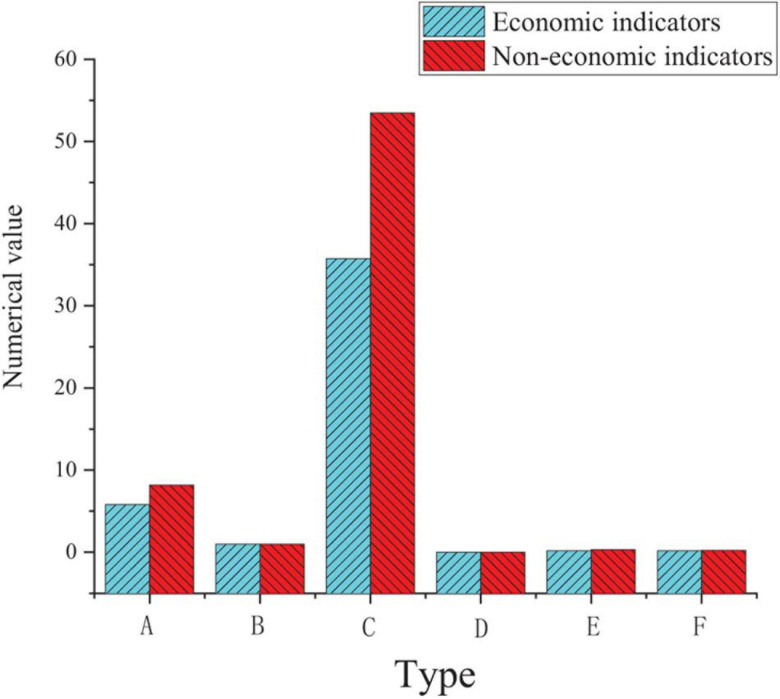
Multiple regression analysis of risk taking and entrepreneurial performance at all levels (A, β value; B, *t* value; C, VIF value; D, F test; F Sig.; E, R^2^; F, △R^2^).

Figure 7 reveals that risk taking has a significant positive impact on the economic indicators of entrepreneurial performance, with F value of 35.738, β value of 0.485, R^2^ value of 0.194 and *P* value of 0.000. Risk taking has a significant positive impact on the non-economic indicators of entrepreneurial performance. The F value is 54.899, β value is 0.520, R^2^ value is 0.312, and *P* value is 0.000. The regression model verifies the hypothesis H2-1 and H2-2.

Table 4 suggests that the vitality, dedication and concentration of work engagement have a significant positive impact on the economic indicators of entrepreneurship performance. The F value is 19.468, β value is 0.327, 0.165 and 0.395, R^2^ value is 0.254, and *P* value is 0.000. The vitality, dedication and concentration of work engagement have a significant positive impact on the non-economic indicator level of entrepreneurial performance. The F value is 19.312, β value is 0.297, 0.186 and 0.194, R^2^ value is 0.283, and *P* is 0.000. The regression model verifies the hypothesis H3-1, H3-2, H3-3, H3-4, H3-5 and H3-6.

**TABLE 4:** Regression analysis of work engagement and entrepreneurial performance at all levels.

		**β value**	**t value**	**VIF value**	**F test**	**Sig.**	**R^2^**	**△R^2^**
		**Economic indicators**						
Work engagement	Vitality	0.327***	3.694	1.943	19.468	0.000	0.254	0.208
	Dedication	0.165***	2.106	2.157				
	Concentration	0.395***	3.064	1.049				
		**Non-economic indicators**						
Work engagement	Vitality	0.297***	4.249	1.356	19.312	0.000	0.283	0.246
	Dedication	0.186***	3.690	1.968				
	Concentration	0.194***		1.785				

*** means *P* < 0.01.

### Multiple Regression Analysis of Entrepreneurial Passion and Risk Taking on Work Engagement

**FIGURE 8:**
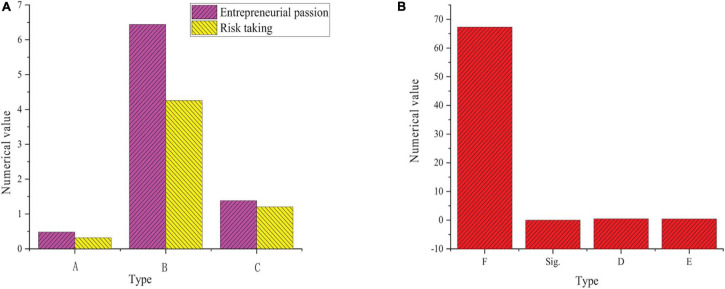
Multiple regression analysis of entrepreneurial passion and work engagement risk taking [**(A)** data analysis data; **(B)** calculation and statistics; A, β value; B, t value; C, VIF value].

The results of multiple regression analysis in Figure 8 show that the F value of entrepreneurial passion and risk taking on work engagement is 67.324, the β value is 0.482 and 0.316, the R^2^ value is 0.476, and the *P* value is 0.000. It means that the regression model reaches a significant level, which verifies the hypothesis H4 and H5.

Table 5 shows that starting passion and founding passion of entrepreneurial passion have a significant positive impact on the vitality of work engagement. The F value is 27.522, β value is 0.298 and 0.325, R^2^ value is 0.273, and *P* value is 0.000. Starting passion and founding passion of entrepreneurial passion have a significant positive impact on the dedication level of work engagement. The F value is 28.274, the β value is 0.346, and 0.287, R^2^ value is 0.285, and *P* value is 0.000. Starting passion and founding passion of entrepreneurial passion have a significant positive impact on the concentration level of work engagement. The F value is 37.435, β value is 0.339, and 0.385, R^2^ value is 0.354, and *P* value is 0.000. The regression model verifies the hypothesis H4-1, H4-2, H4-3, H4-4, H4-5, and H4-6.

**TABLE 5:** Multiple regression analysis of entrepreneurial passion and work engagement at all levels.

		**β value**	**t value**	**VIF value**	**F test**	**Sig.**	**R^2^**	**△R^2^**
		**Vitality**						
Entrepreneurial passion	Starting passion	0.298***	3.457	1.200	27.522	0.000	0.273	0.252
	Founding passion	0.325***	4.134	1.200				
		**Dedication**						
Entrepreneurial passion	Starting passion	0.346***	4.232	1.200	28.274	0.000	0.285	0.263
	Founding passion	0.287***	3.627	1.200				
		**Concentration**						
Entrepreneurial passion	Starting passion	0.339***	4.295	1.200	37.435	0.000	0.354	0.312
	Founding passion	0.385***	3.972	1.200				

*** means *P* < 0.01.

### The Mediating Effect of Work Engagement

In order to verify the mediating effect of work engagement on entrepreneurial passion and risk taking on entrepreneurial performance, the mediating effect analysis process is adopted. Model 1: the independent variable has significant standard regression coefficient to the dependent variable. Model 2: the independent variables have significant standard regression coefficients to the intermediate variables. Model 3: there is a significant standard regression coefficient between mediators and dependent variables. Mode 4: the relationship between independent variables and dependent variables will weaken due to the existence of mediators, or even form an insignificant situation. However, the relationship between mediators and dependent variables is still significant.

The above four models can verify whether the mediating effect of mediators exists. If the β value between the independent variable and dependent variable of mode 4 is lower than that of mode 1, and the relationship is not significant, it means that the mediator has a complete mediating effect; if the relationship is significant and decreases, it means that the mediator has a partial mediating effect. Therefore, the results of the above regression analysis will be integrated to explore the mediating effects of entrepreneurial passion, risk taking and entrepreneurial performance.

Table 6 reveals that entrepreneurial passion has a significant impact on entrepreneurial performance. Entrepreneurial passion reaches a significant level for work engagement (Work engagement has a significant impact on entrepreneurial performance). The overall explanation of entrepreneurial passion is (R^2^ = 38.7%), while ΔR^2^ = 5.8% has a slight increase compared with mode 1. The comparison of the four modes suggests that the β value of entrepreneurial passion decreases from 0.571 to 0.371, and reaches the significant level. The indirect effect produced by entrepreneurial passion through work engagement is 0.346, and the total effect is 0.917. According to the evaluation criteria, it shows that work engagement has a partial mediating effect on entrepreneurial passion and entrepreneurial performance, which verifies the hypothesis H6.

**TABLE 6:** Mediating effects of work engagement.

**Research variables**	**Entrepreneurial performance**		**Work engagement**	
	**Mode 1**	**Mode 3**	**Mode 4**	**Mode 2**
Entrepreneurial passion	0.571***		0.371***	0.627***
Work engagement		0.551***	0.318***	
F test	72.493	65.391	47.424	98.635
Sig.	0.000	0.000	0.000	0.000
R^2^	0.326	0.304	0.387	0.394
Adj-R^2^	0.321	0.299	0.379	0.390
△R^2^			0.058	

*** means *P* < 0.01.

To sum up, entrepreneurial passion and risk-taking, work engagement and entrepreneurial performance are significantly positively correlated; risk-taking spirit is positively correlated with job engagement and entrepreneurial performance, and work engagement and entrepreneurial performance are also significantly positively correlated. The results of multiple regression analysis show that entrepreneurial enthusiasm, risk-taking and work engagement are correlated with entrepreneurial performance, which verifies hypothesis H1, H2 and H3; the regression analysis structure of entrepreneurial passion and entrepreneurial performance at all levels verifies the hypothesis H1-1, H1-2, H1-3, and H1-4. The results of multiple regression analysis of risk-taking and entrepreneur performance at all levels verify the hypothesis H2-1 and H2-2; the regression analysis results of work participation and entrepreneur performance at all levels verify the hypothesis H3-1, H3-2, H3-3, H3-4, H3-5, and H3-6; the results of multiple regression analysis of entrepreneurial passion and job risk-taking verify hypothesis H4 and H5; the results of multiple regression analysis of entrepreneur enthusiasm and work engagement at all levels verify the hypothesis H4-1, H4-2, H4-3, H4-4, H4-5, and H4-6; the mediating role of work participation is evaluated to verify hypothesis H6. Therefore, the correctness of the hypothesis proposed is proved through several confirmatory tests, and the relationship among different influencing factors is proved.

## Conclusion

This exploration is primarily to study the influencing factors of entrepreneurial performance and solutions to legal problems based on entrepreneurial passion psychology. In the research program, entrepreneurial passion and risk-taking are the antecedent variables, and work engagement is the mediator. Based on this theoretical model, entrepreneurial performance is discussed. The empirical results show that entrepreneurial passion exerts a significant positive impact on entrepreneurial performance. Risk taking and work engagement exert a significant positive impact on entrepreneurial performance. Entrepreneurial passion exerts a positive and significant impact on work engagement. Entrepreneurial passion and risk-taking behavior of entrepreneurs will affect entrepreneurial performance through work engagement. Those with enthusiastic personality traits have higher learning willingness, higher job satisfaction and engagement. The empirical results will contribute to the theoretical construction of entrepreneurial performance and the antecedents of work engagement. The research results can provide a reference for the development of related theories and make the whole entrepreneurship research theory more complete. However, there are also some shortcomings. The hypothesis is based on the relevant research literature, so the relationship among the influencing factors will not be well displayed. The content of the hypothesis will be further adjusted and optimized in the follow-up study.

## Data Availability Statement

The raw data supporting the conclusions of this article will be made available by the authors, without undue reservation.

## Ethics Statement

The studies involving human participants were reviewed and approved by Tongji University Ethics Committee. The patients/participants provided their written informed consent to participate in this study. Written informed consent was obtained from the individual(s) for the publication of any potentially identifiable images or data included in this article.

## Author Contributions

All authors listed have made a substantial, direct and intellectual contribution to the work, and approved it for publication.

## Conflict of Interest

The authors declare that the research was conducted in the absence of any commercial or financial relationships that could be construed as a potential conflict of interest.

## Publisher’s Note

All claims expressed in this article are solely those of the authors and do not necessarily represent those of their affiliated organizations, or those of the publisher, the editors and the reviewers. Any product that may be evaluated in this article, or claim that may be made by its manufacturer, is not guaranteed or endorsed by the publisher.
